# Botanical Report

**Published:** 1813-12

**Authors:** 


### BOTANICAL REPORT.

THERE has been so little of novelty in the botanical world for some months past, that we have met with nothing sufficiently interesting to rouse us from our lethargy; and we have suffered the usual period to pass over again and again without furnishing a report on the subject.

The BOTANICAL MAGAZINE has indeed continued to be regularly published, and many very curious plants have been there described and figured; English Botany too has eked out its dribblets; but this long-beaten track we have not felt disposed to tread over, expecting some inducement to deviate into a more interesting path.

At length a new work has appeared, which, for its importance, and more especially for the excellency of its execution, deserves to be particularly noticed; we shall offer no apology for making it the subject of the present report. It is entitled

FERDINANDI BAUER *illustrationes Floræ Novæ-Hollandiæ sive icones generum quæ in Prodromo Floræ Novæ-Hollandiæ et Insulæ Van-Diemen, descripsit Robertus Brown.* This work is intended to consist of figures of all the genera described by Mr. Brown, in his Flora Novæ-Hollandiæ; of which, as yet, only a part of the Prodromus has been made public. In general one species only in each genus will be figured, except when the genus shall consist of several species, divided by the author into two or more natural sections, the different habit or structure of which may require illustration, when the necessary examples will be given. No letter-press will accompany the plates, but with the first number is given a general table, to which the letters, figures, and marks on each plate refer. Thus, by always preserving the same characters to denote similar parts, one general table of explanation serves for the whole; a mode which is attended with this farther advantage, that any person studying the genera from these plates will soon acquire a prima-facie knowledge of all the characters, and will no longer find it necessary to refer to any explanation.

Description is supposed to be rendered unnecessary, by the reference, always engraved on the plate, to Mr. Brown's work, where it is to be expected that every necessary information will be found. At present, however, a part only of the Prodromus Floræ Novæ-Hollandiæ is published; but it is to be hoped that the remainder will not be much longer held from the botanical world. When the larger work, of which this may be considered as the herald, shall appear, more ample details may be expected. But should no more than the Prodromus ever see the light, when this shall be completed the botanical reader will not much feel the want of a more copious history.

But, to return to the ILLUSTRATIONS. The drawings, we are informed in the Preface, have been, for the most part, made from the living plants in their native soil; for Mr. Bauer and Mr. Brown both accompanied Captain Flinders on his voyage round the Coasts of New-Holland, in the years 1802, 3, 4, and 5. For some of the genera, not detected in this voyage, Mr. Bauer is indebted to the collection of drawings made under the direction of Sir Joseph Banks, during Captain Cook's first voyage round the world; and some few will necessarily be taken from dried specimens, preserved in the herbarium of this botanist, or in that of Mr. Brown.

The

The first number consists of the five following articles:

1. Johnsonia *lupulina*. Brown Prod. page 287.
2. Pterostylis *grandiflora*. Ibid, page 327
3. Banksia *coccinea*. Ibid, page 394.
4. Chloanthes *stœchadis*. Ibid, page 514.
5. Stylidium *violaceum*. Ibid, page 569.

The drawings are executed in the first style. The whole plant, or such portion of it as the size of a large folio plate will admit of, is represented of the natural size, and dissections of the parts of fructification, in almost every point of view, are added. Many will, we apprehend, think that these have been multiplied even beyond what is necessary. But when it is recollected that Mr. Bauer is not only a draughtsman of the very first order, but is likewise an excellent botanist; and that these dissections are made, the drawings taken, and the engravings executed by the same hand; the botanical student will feel very grateful for the pains that have been taken to display the minute organs of fructification in such a conspicuous manner, that the structure of the different genera may perhaps be better studied from these representations, than from the plants themselves; for very few persons are capable of making such dissections, and adapting them to the microscope, so as to display the various parts in so lucid a manner; and to those that can, the value of the time spent in such enquiries, will be no mean consideration.

Certain persons who value these things more as pictures than as illustrations of the science of botany, may probably consider the plate as too crouded, from the number of these dissections; and we are not sure but that our artist would have acted more wisely, that is, more consonant to his own interest, had he put these dissections into a separate plate, by which means the beautiful representations of the plant would have stood distinct and unincumbered.

We hope that Mr. Bauer will meet with due encouragement to proceed with his illustrations, which, when completed, will, in our opinion, outrival the most celebrated botanical works, that are now carrying on upon the continent. The circumstances of the times, it must be allowed, are not very favorable to the prosecution of works of this nature; yet it would be a disgrace to the nation, if this excellent artist should not be able to proceed for want of encouragement, whilst, under similar difficulties, not one only, but several, more expensive works are carrying on at the same time in Paris; none of which, however, in faithfulness of representation and accuracy of dissection, can vie with the one which we are now recommending. The price of the number colored is one guinea and a half, and only five shillings plain. These prices seem a little disproportionate, but the colored copies may really be considered as cheap, when the high style in which they are finished, hardly exceeded by the drawings themselves, is taken into the account.

They are dissected, drawn, and engraved, by the same hand, a circumstance of great moment to insure faithful imitation; the coloring of the present number is executed in so masterly a manner, that we cannot but suspect that Mr. Bauer himself has at least put a finishing hand to them.

The fourth and fifth volume of the Hortus Kewensis, which finishes the work, have been some time printed; we fear the publication is delayed by Mr. Aiton's indisposition.

